# AFRP Influence on Parallel Bamboo Strand Lumber Beams

**DOI:** 10.3390/s18092854

**Published:** 2018-08-29

**Authors:** Huizhong Zhang, Haitao Li, Ileana Corbi, Ottavia Corbi, Gang Wu, Chengjie Zhao, Tongwei Cao

**Affiliations:** 1 College of Civil Engineering, Nanjing Forestry University, Nanjing 210037, China; zhanghuizhong1993@njfu.edu.cn (H.Z.); chengjiezhao@njfu.edu.cn (C.Z.); tongweicao@njfu.edu.cn (T.C.); 2 Key Laboratory of Concrete and Pre-Stressed Concrete Structure of Ministry of Education, Southeast University, Nanjing 210096, China; g.wu@seu.edu.cn; 3 Department of Structures for Architecture and Engineering, University of Naples Federico II, via Claudio 21, 80133 Napoli, Italy; ileana.corbi@unina.it (I.C.); ottavia.corbi@unina.it (O.C.)

**Keywords:** AFRP, strain measuring, laser displacement sensors, damage identification, parallel bamboo strand lumber beams, composite structures

## Abstract

The mechanical properties of parallel bamboo strand lumber beams could be improved by aramid fiber reinforced polymer (AFRP). So far, no investigation has been conducted on the strengthening of engineering bamboo beams using AFRP. In order to study the efficiency of AFRP reinforcement on parallel bamboo strand lumber beams, 13 beams had been tested and analyzed. Strain gauges and Laser Displacement Sensors were used for the tests. By sensing the strain and deformation data for the specimens under the applied loads, the results showed that AFRP can effectively improve the flexural mechanical properties of parallel bamboo strand lumber beams. However, this reinforcement cannot increase the deflection of bamboo beams indefinitely. When the cloth ratio was 0.48, the deflection of the specimens reached its maximum. With the increase of cloth ratio, the stiffness of parallel bamboo strand lumber beams was increasing. When the cloth ratio reached 0.72%, compared with the un-reinforced specimen, the stiffness increased by 15%. Therefore, it can be inferred that bonding AFRP on the considered specimens can increase the stiffness of parallel bamboo strand lumber beams. The ductility of the specimen can be effectively enhanced by adopting the AFRP provision.

## 1. Introduction

Due to its high strength, light-weight, renewable, and environment-friendly characteristics, bamboo has attracted the attention of many scholars [1,2,3,4,5,6,7,8,9,10,11,12,13,14,15,16,17,18,19,20,21,22]. Bamboo culm can be disassembled into long bamboo strands [23,24,25,26,27,28]. After some treating processes, all strands can be put into molds and pressed into parallel bamboo strand lumber (PBSL) [23,24,25,26,27,28,29,30,31,32,33,34,35,36]. Due to its high strength, uniform material, and good processing performance, more and more experts and scholars [23,24,25,26,27,28,29,30,31,32,33,34,35,36] have carried out experimental research on it.

The mechanical properties for PBSL have been studied by many scientists [23,24,25,26,27,28,29,30,31,32,33,34,35,36]. Wang et al. [23] have researched the control system of automatic bamboo scrimber cold pressing units. Yu et al. [24] have studied the fabrication, material properties, and application of PBSL. Gong et al. [25] investigated the tensile and compressive strength of PBSL. Shangguan et al. [26] proposed strength models for PBSL under compression and they also studied the effects of heat treatment on the properties of PBSL [27]. Kumar et al. [28] investigated the influence of density on the mechanical and water absorption properties. Xu et al. [29] studied the compressive and tensile properties of a bamboo scrimber at elevated temperatures. Yu et al. [30] studied the preparation, physical, mechanical, and interfacial morphological properties of PBSL. Sharma et al. [31] investigated the basic mechanical properties of PBSL. Zhong et al. [32] examined the design value of the compressive strength for bamboo fiber-reinforced composite based on reliability analysis. The effect of eccentricity ratio properties of 50 (PBSL) column specimens of parallel bamboo strand lumber columns under eccentric compression was measured by Li et al. [33]. Zhong et al. [34] investigated the bending properties of newly designed reinforced bamboo scrimber composite beams. The results show that eccentricity ratio is the most important factor affecting the bearing capacity of columns. Su et al. [35] investigated the mechanical properties and ductility failure of parallel bamboo strand lumber columns under axial compression. Zhong et al. [36] studied the compressive strength of bamboo reconstituted wood at different temperatures and provided the basis for fire prevention of bamboo materials. 

In recent years, FRP has been widely used in the field of civil engineering [37,38,39,40] because of its advantages of high strength, corrosion resistance, light-weight, simple construction, and so on. Many experts began to use FRP to strengthen many kinds of materials [39,40,41,42,43,44,45]. Wang [41] tried to strengthen double-edged cracked steel plates by FRP. Liu [42] presented a crack monitoring method for an FRP-strengthened steel structure deploying a microstrip antenna sensor. Tang [43] developed a new type of self-sensing fiber reinforced polymer (FRP) bar. In order to make better use of FRP, many new methods are provided for monitoring of FRP Structures [42,43,44,45,46,47]. Many experts and scholars also began to use FRP cloth to strengthen the mechanical properties of bamboo reconstituted wood. Using different FRP types, the mechanical buckling properties of glued wood columns with different slenderness ratios were tested and studied by Taheri [47]. Based on Euler's formula, the formula for calculating yield load of FRP confined glued wood columns was inferred. Research results showed that the compressive strength and stiffness of glued wood columns could be improved by FRP fiber cloth. Awaludin et al. [48] chose FRP fiber cloth and natural fiber to strengthen bamboo joints. Considering the effect of different loading directions, the mechanical properties of the joints have been examined through experimental study. Qin [49] studied the bending behavior of BFRP composite bamboo beams. Zhang [50] investigated the mechanical behavior of wood beams strengthened with carbon fiber sheets. 

Up to now, although more and more experts are paying attention to FRP PBSL beams, their study is still limited, particularly for AFRP reinforced beams. Considering the cloth ratio, this paper aims to investigate how AFRP influences the mechanical properties of PBSL beams.

## 2. Materials and Methods

### 2.1. The Specimens

**Figure 1:**
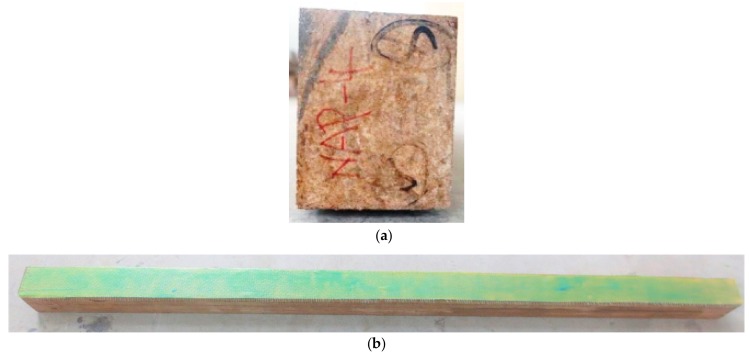
Parallel bamboo beam specimens. (**a**) is the cross section; (**b**) is the bottom of specimen with AFRP.

With the size of 80 mm × 100 mm × 1860 mm, 13 beam specimens were designed considering AFRP cloth ratios. Detailed information can be seen from Table 1. The cross section for the beam can be seen from Figure 1a. PBSL was made from Jiangxi Feiyu Bamboo Group Co. Ltd. (Fengxin, China). With a thickness of 0.193 mm, AFRP were bought from Carbon Composites Materials Co. Ltd. from Tianjin, China. The test tensile strength and elastic modulus of AFRP were 1893 MPa and 2023 GPa, respectively. The specimens are divided into four groups according to the layer of AFRP. No AFRP cloth was pasted for Group NAP. As for LAP1, LAP2, and LAP3, 1 layer, 2 layers, and 3 layers were pasted on the bottom of each group, respectively. AFRP covered the whole bottom surface as show in Figure 1b.

### 2.2. Test Design

**Figure 2:**
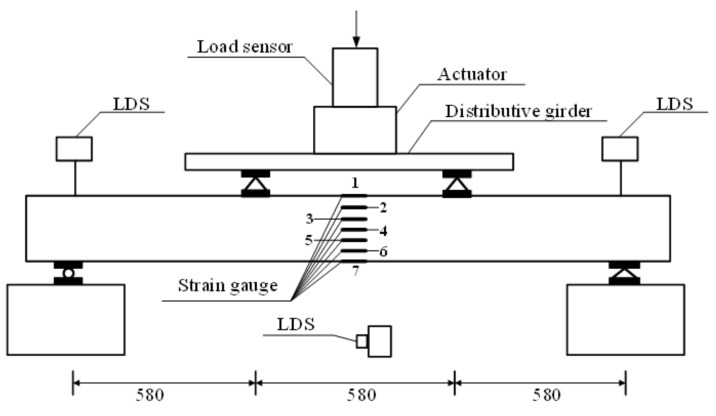
Test setup.

The test setup is shown in Figure 2. The loading system was designed according to the GB/T50329-2012 Standard for test methods of wood structure. A 100 kN electro-hydraulic servo testing machine produced by Hangzhou Bangwei Electromechanical Control Engineering Co. Ltd. (Hangzhou, China) was adopted for the testing. Data acquisition was carried out by TDS-530 static data acquisition instrument with acquisition precision of 0.1 με. The static monotonic loading method was chosen, which combines load control with displacement control. The load was controlled by the load before 80% of the ultimate load, then the displacement control started until the end of the loading. The loading speed did not exceed the calculated value from formula (1) [51].

(1) $$v=5\times {10\;}^{-5}\times \frac{a}{3h}\left(3l-4a\right)$$

Among them: *a* is the distance between the beam support point and the nearest loading point, *h* is the height of the specimen, and *l* is the calculation length of the beam specimen.

The arrangement of the strain gauges is shown in Figure 2. Seven strain gauges (numbered from top to bottom) were installed along the height of the cross section in the middle span of the specimens. Three laser displacement sensors (LDS) were used to measure the displacement of the two beam supports and the middle deflection. The applied load is measured by a load sensor which is connect to the actuator.

## 3. Test Results and Analysis

### 3.1. Damage Patterns and Analysis

#### 3.1.1. PBSL Beams

**Figure 3:**
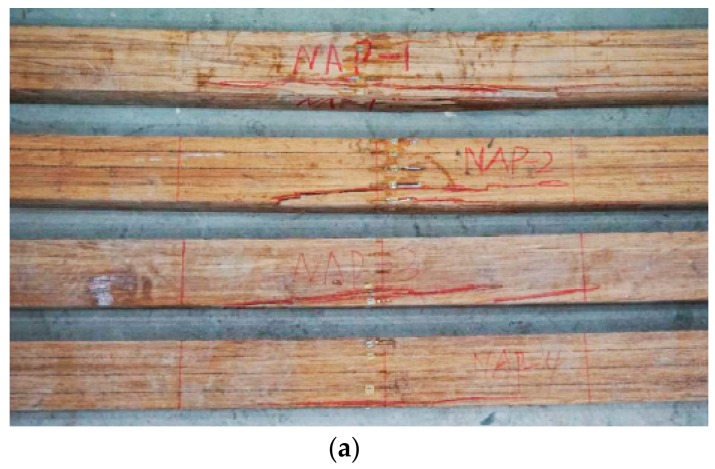

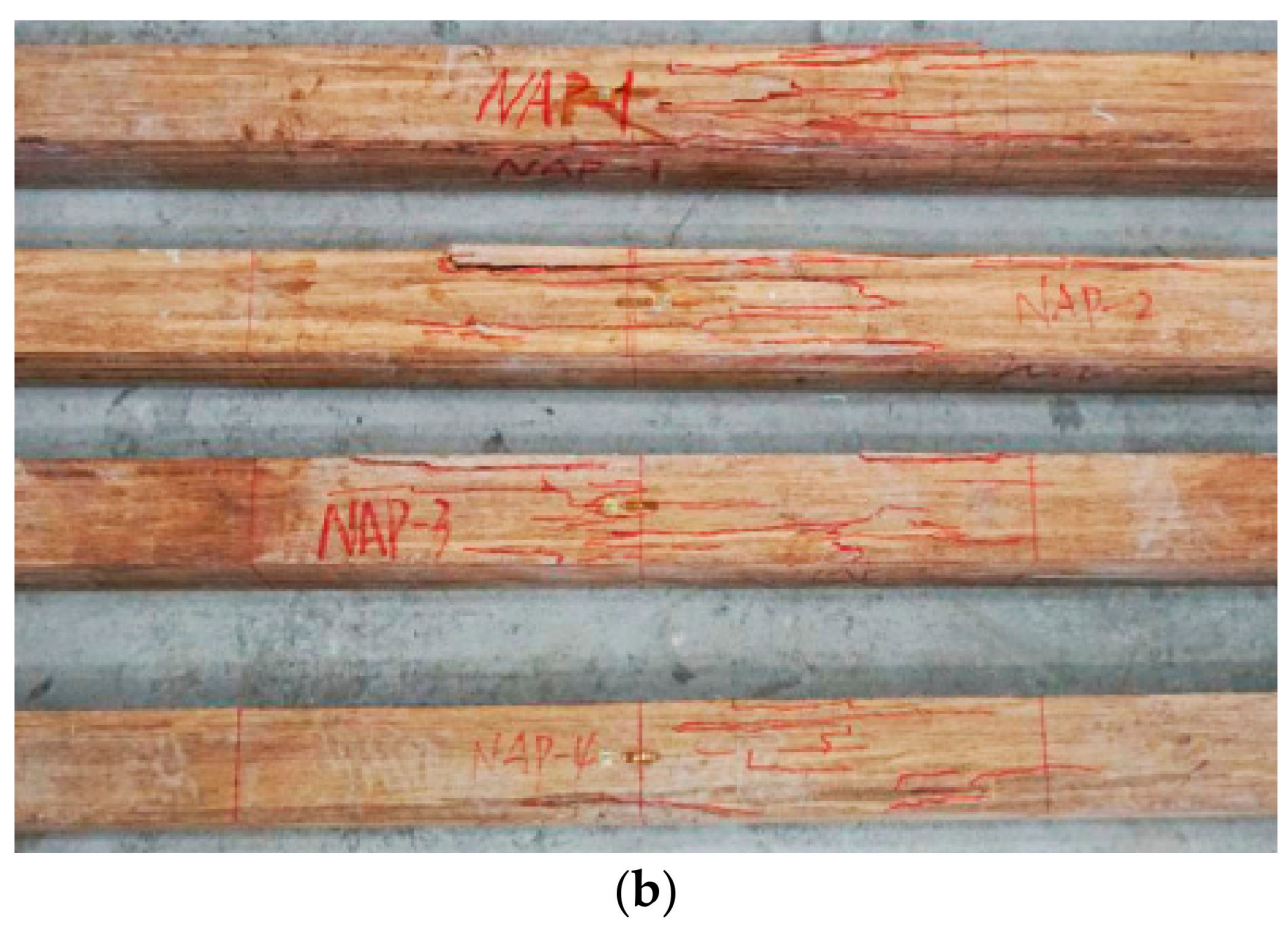
This is the failure photos for parallel bamboo strand lumber (PBSL) beams: (**a**) is the side surfaces; (**b**) is the bottom surface.

Failure photos on the side surfaces and bottom surfaces are shown in Figure 3. All specimens experienced elastic stage, elastic-plastic stage, and failure stage. Typical bending failure happened for all four beams. The first crack always appeared on the bottom surfaces in the pure bending area for the beams. Natural bamboo joints are the main reason for the first cracks.

#### 3.1.2. AFRP Strengthened PBSL Beams

**Figure 4:**
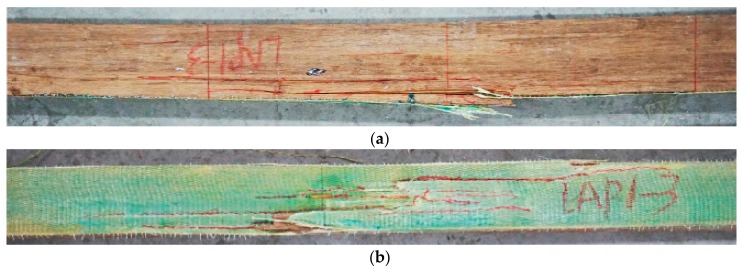
This is the typical failure photos for mode I (LAP1-3): (**a**) side surface; (**b**) bottom surface.

Three typical failure modes were classified for AFRP strengthened PBSL beams. Figure 4 shows typical failure photos for mode I. Both the AFRP and bamboo fibers broke at the bottom of the beam. Taking LAP1-3 as an example, there was no evident phenomenon at the initial loading stage. With the increase of load, a slight noise appeared when the load value was 51.2 kN and the deformation was evident for the beam. After a short while, the first crack for AFRP appeared in the middle pure bending area. As the loading value became bigger and bigger, more and more cracks appeared in the pure bending part. AFRP cloth was pulled off little by little. The loading value decreased quickly after the peak load value of 57.5 kN with displacement of 55.6 mm and was finally crushed. There was no evident crack on the compression surface during the whole loading process. This failure mode was due to the fact that the ultimate tensile strain of bamboo fiber was smaller than the ultimate tensile strain of AFRP cloth in general. A sudden breaking of bamboo fibers caused a certain retraction, which made the stress of AFRP cloth suddenly increase and reached the ultimate tensile strain. Thus, the damage was produced. This failure mode also happened to AP2-2.

**Figure 5:**
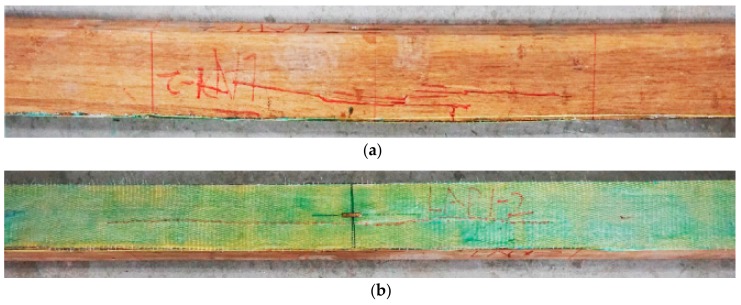
This is the typical failure photos for mode II (LAP1-2): (**a**) side surface; (**b**) bottom surface.

Typical failure photos for side surface and bottom surface for mode II are shown in Figure 5. Only one specimen (LAP1-2) belongs to this failure mode. At the beginning of the test, there was no evident phenomenon in the specimen. As the load increased, the first noise appeared with the load value of 44 kN. The first crack appeared in the inner part of PBSL beam but not the AFRP sheet. With the increase of load, more and more cracks happened in the inner part of the beam. The AFRP sheet split longitudinally along the beam length in the middle span when the load value was close to the ultimate load. After achieving the ultimate load 45.4 kN with the displacement of 48.5 mm, the specimen was crushed suddenly with rapid decreasing of the load. There was no evident crack on the compression surface.

**Figure 6:**
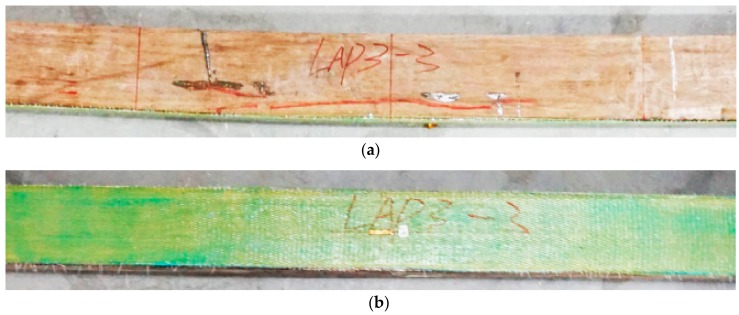
This is the typical failure photos for mode III (LAP3-3): (**a**) side surface; (**b**) bottom surface.

**Figure 7:**
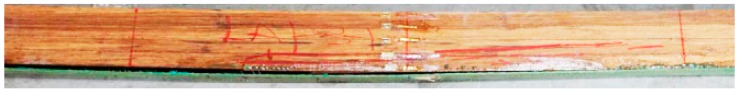
Failure photos for LAP3-1.

Figure 6 shows typical failure photos for mode III. LAP3-3 was chosen as an example to illustrate the failure process. Similar to the other specimens, no evident failure phenomenon except the deformation could be seen during the elastic stage. A slight noise could be heard with the loading value of 46.7 kN. Cracks appeared firstly in the inner part of PBSL but no clear failure phenomenon could be seen for AFRP sheet. After the peak load value of 67.6 kN with the displacement of 70.8 mm, the beam was crushed suddenly. After finishing the loading, no cracks can be seen for AFRP cloth but the glue between AFRP and bamboo doesn’t work in the middle one third part of the beam. The 5 other specimens failed similarly as LAP3-3 which are LAP1-1, LAP2-1, LAP2-3, LAP3-2, and LAP3-3. While for LAP3-1, the failing process is similar as mentioned above and the only difference is that most part of AFRP sheet is separated from the beam as shown in Figure 7.

As discussed above, it was noticed that the specimens with three layers of AFRP began to break when the loading value was about 90–99% of the ultimate load, and the specimens with two layers of AFRP began to break at 86–98% of the ultimate load. While the specimens with one layer of AFRP started to fail when the load was about 71–91% of the ultimate load. However, cracks appeared firstly when the load value was about 64–91% of the ultimate load for the specimens without AFRP. AFRP cloth could effectively improve the mechanical performance of PBSL beams, and the initial failure load increases with the increase of cloth ratio.

### 3.2. Results of the Test

The AFRP cloth ratio for the beams could be calculated as the following [51].

(2) $$\rho =\frac{A_{S}\;}{bh}$$

*A*_S_ is the cross-sectional area of the AFRP cloth, *b* and *h* are the width and height for the beam cross-section, respectively.

The strength of the beams could be calculated by the following formula [51].

(3) $$f_{m}=\frac{aF_{u}\;}{2W}$$

*a* is the distance from the loading point to the nearest beam support (the values for all specimens are 580 mm), *F*_u_ is the ultimate load for the specimen (N), and *W* is the section modulus of the specimen (mm^3^).

The flexural rigidity *EI* for the specimens could be calculated by the following equation [51].

(4) $$EI=\frac{a\Delta F\;}{48\Delta \omega }(3L^{2}-4a^{2})$$

*E* is the experimental modulus of elasticity before the proportional ultimate load, *I* is the cross section moment of inertia of the specimen (mm^4^), ∆*F* is the load increment of elastic stage (N), ∆*ω* is the deflection increment in span corresponding to load increment in elastic stage (mm), and *L* is specimen span (mm).

Based on the designed parameters for the beam specimens, *a* = *L*/3, formula 4 could be simplified as the following.

(5) $$EI=\frac{23PL^{3}\;}{1296\omega }$$

*P* is the proportional limit load (N), *ω* is the displacement in the middle span corresponding to the proportional ultimate load of the specimen (mm).

The ductility coefficient $\mu_{\Delta }$ could be calculated according to Formula (6) [51].

(6) $$\mu_{\Delta }=\frac{w_{0.85\;}}{w_{u}}$$

$w_{0.85}$ is the mid-span displacement when the load value falls to 85% of the ultimate bearing capacity, $w_{u}$is the mid-span displacement of specimens for the ultimate bearing capacity.

The test data were summarized and sorted, and the results are shown in Table 2.

**Figure 8:**
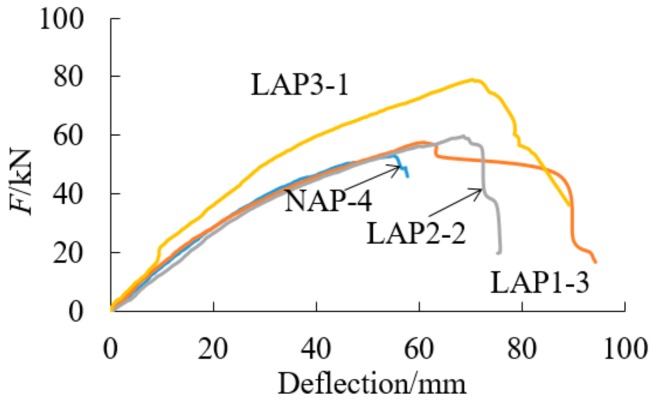
Load–deflection curve.

**Figure 9:**
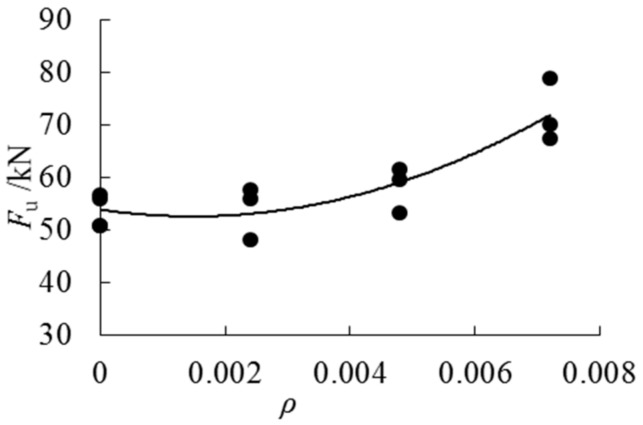
Ultimate load–cloth ratios trend curve.

It can be seen from Table 2 and Figure 8 and Figure 9 that AFRP cloth can effectively increase the bending properties of parallel bamboo strand lumber beams. With the increase of the cloth ratio, the ultimate flexural capacity is also increased. The average ultimate load of the unreinforced parallel bamboo strand lumber beams is 53.7 kN. The ultimate bearing capacity of the composite beams with three layers of cloth is larger than that of the beams with two layers of cloth and the beams with one layer of cloth, which are larger than those with a non-adhesive cloth. When the cloth ratio is 0.24%, the bearing capacity is increased by 0.6%, and when the cloth ratio is 0.48%, the bearing capacity is increased by 10.8%. When the cloth ratio is 0.72%, the bearing capacity is increased by 34.5%. It can also be observed from the data that the ultimate bearing capacity of LAP1-2 is extremely low, which is due to the inherent damage in the beam. 

**Figure 10:**
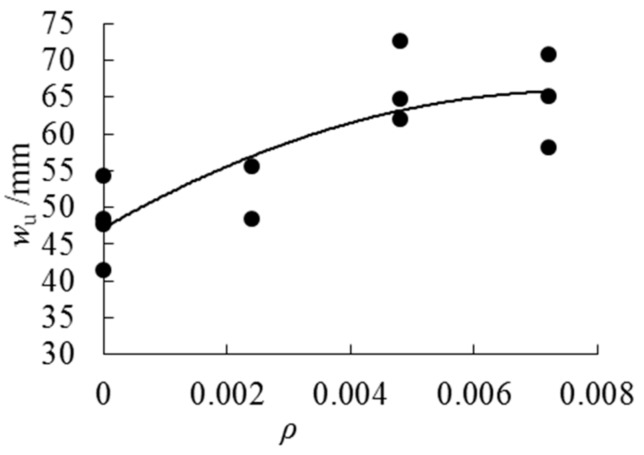
Ultimate deflection–cloth ratios trend curve.

As can be seen from Figure 10, the ultimate deflection increases obviously with the increase of cloth ratio when the cloth ratio is less than 0.48%. The deflection of the specimens with a cloth ratio of 0.48% and a cloth ratio of 0.72% is similar. It is proved that AFRP cannot increase the deflection of the beam indefinitely. 

**Figure 11:**
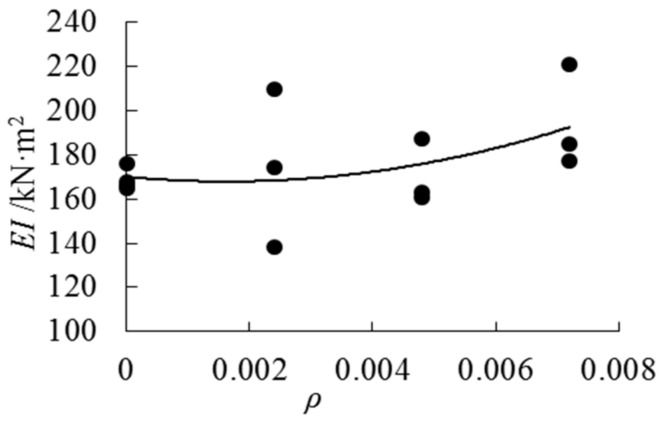
Stiffness–cloth ratios trend curve.

Figure 11 shows the variation trend for the flexural rigidity values with the increase of cloth ratios. Overall, the flexural rigidity increases when the cloth ratio increases. Group LAP1 has the largest discreteness among the four groups. When the cloth ratio reaches 0.72%, the mean flexural rigidity value of the specimen increases by 15% compared with the beams without AFRP cloth. It is concluded that bonding AFRP could improve the stiffness of the beam. Based on the test results, the following formula can be used to express the relationship between flexural rigidity and AFRP cloth ratio.

(7) $$EI=794793\rho^{2}-2602\rho +170,\;(0\leq \rho \leq 0.0072),\;(R^{2}=0.2071)\;$$

*E* is the elastic modulus, *I* is the moment of inertia of the cross section of the specimen (mm^4^), *ρ* is the cloth ratio for AFRP.

**Figure 12:**
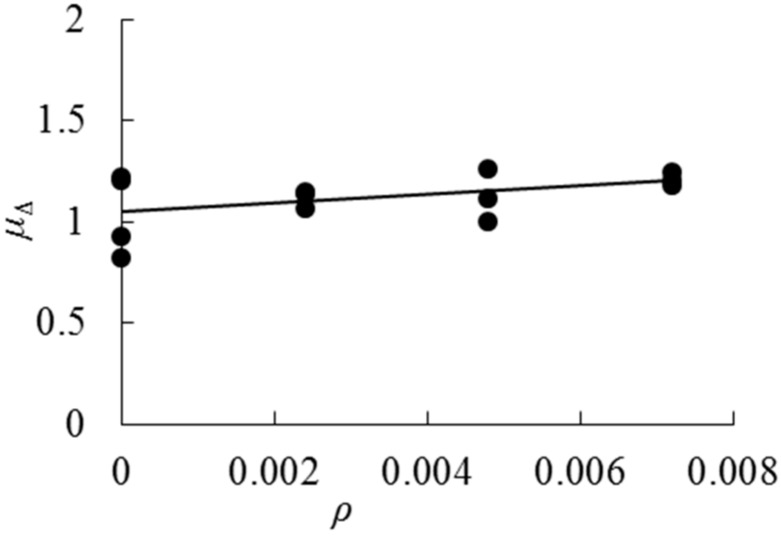
Ductility factor–cloth ratios trend curve.

Figure 12 plots how the values of ductility change with the increase of cloth ratios. It can be seen that the ductility values become bigger and bigger with the increase of cloth ratios. AFRP cloth can enhance the ductility of the beams. The relationship between the ductility coefficient and the cloth ratio could be expressed as follows.

(8) $$\mu_{\Delta }=21.471\rho +1.0486,\;\left(0\leq \rho \leq 0.0072),\;(R^{2}=0.2153\right)$$

***μ***_Δ_ is the ductility coefficient, *ρ* is AFRP cloth ratio.

Table 3 shows the mid-span displacement comparison under different load values for all four groups. As it can be noticed from Table 3, under the same load, the mid-span displacement of the AFRP PBSL beams is smaller than that for PBSL beams, which indicates that AFRP plays a certain role in strengthening the stiffness of the specimen. Especially for group LAP3, the average mid-span displacement of the specimens is obviously smaller than that of other specimens.

### 3.3. Load-Strain Curves Comparison

**Figure 13:**
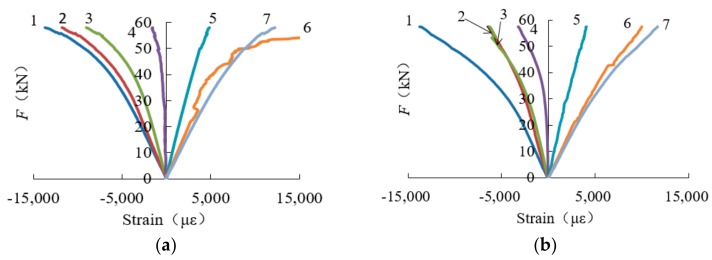

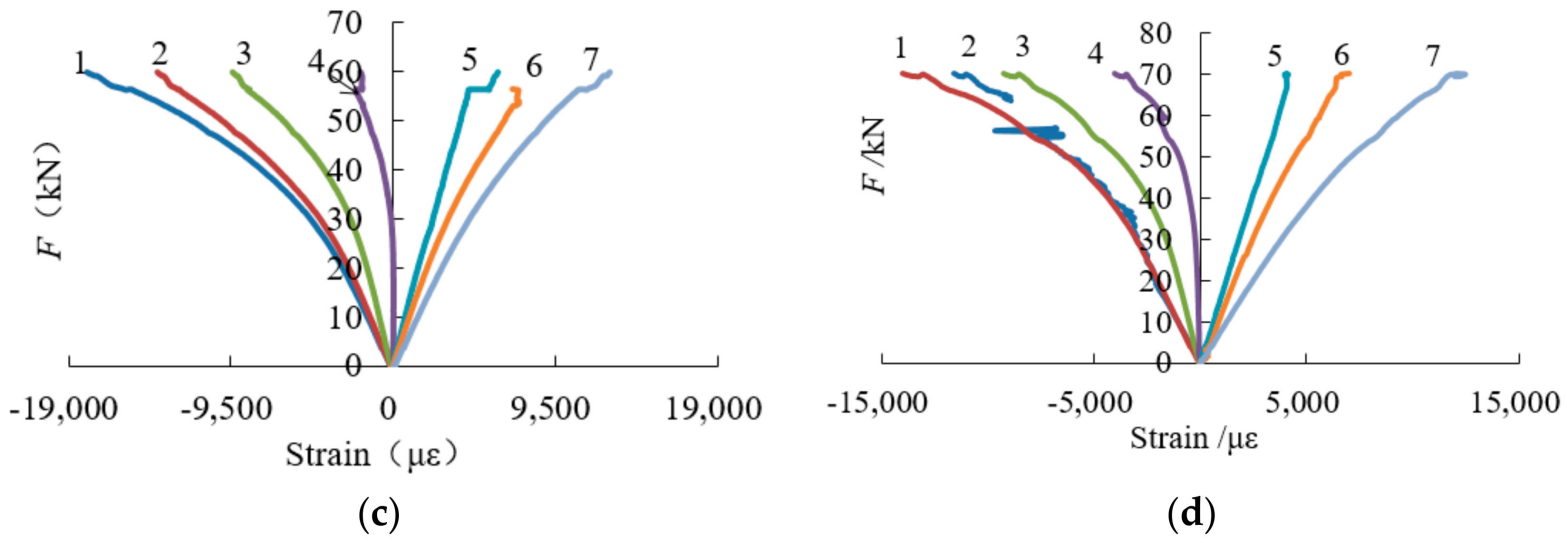
Typical load–strain curves for four groups: (**a**) load–strain curves for specimen NAP-2; (**b**) load–strain curves for specimen LAP1-3; (**c**) load–strain curves for specimen LAP2-2; (**d**) load–strain curves for specimen LAP3-2.

Typical load–strain curves for each group are shown in Figure 13. The load–strain curves both for the tensile and compression side of the beam are basically linearly changed during the elastic stage. As the load values become bigger and bigger, the curves belong to the compression side reaches the elastic stage limit earlier compared with the tensile side. As loading continues, the strain curves under compression enter the elastic-plastic stage, and line 4 for the strain values in the central line starts to curve as the middle strain gauge in the side surface begins to undertake compression strain. This means that the neutral axis begins to move downward.

### 3.4. Plane Cross Section Strain

**Figure 14:**
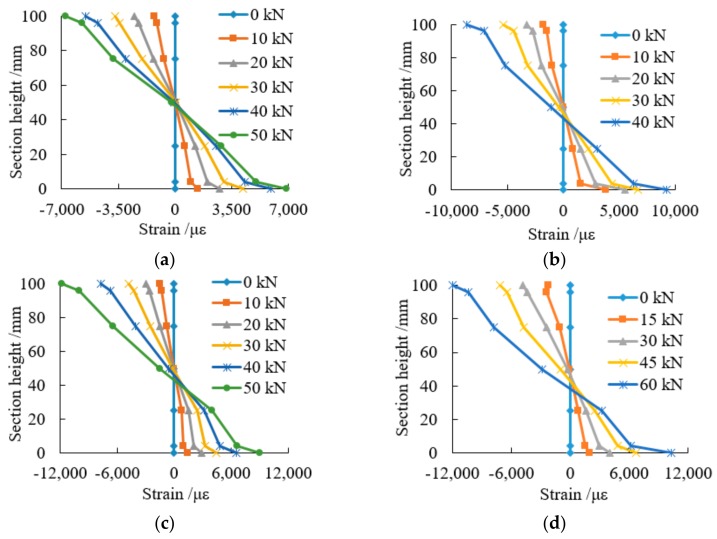
Strain of cross section of typical specimens: (**a**) strain of cross section of NAP-3; (**b**) strain of cross section of LAP1-2; (**c**) strain of cross section of LAP2-2; (**d**) strain of cross section of LAP3-3.

Figure 14 shows how the strain values change along the cross-sectional height with the increase of load. It can be observed that the cross section strain for the beams shows an approximately linear distribution along the height direction, meaning the specimen basically accords with the assumption of the plane section.

**Figure 15:**
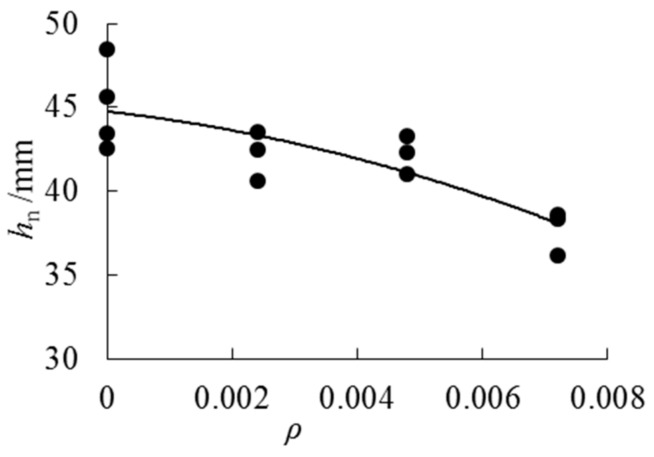
The relationship between the neutral axis height and cloth ratio.

The neutral axis position for all specimens is shown in Table 4, and Figure 15 plots how the neutral axis changes with increasing cloth ratio. It can be noticed that when the specimen reaches the ultimate load, all the neutral axis height values are less than 50 mm (half of the beam height), which means that the neutral axis moves downward. The neutral axis of the specimen with AFRP cloth moves down more than the specimens without AFRP cloth, and as the number of layer increases, the neutral axis decreases. This phenomenon shows that the AFRP cloth plays a certain role in the pulling force. The more the neutral axis goes down, the bigger the pulling force AFRP cloth will bear.

On the basis of the test results, the following expression can be used to describe the relationship between neutral axis height and cloth ratio.

(9) $$h_{n}=-69573\rho^{2}-426.49\rho +44.759,\;\left(0\leq \rho \leq 0.0072),\;(R^{2}=0.6817\right)$$

*h_n_* is the neutral axis height, *ρ* is the cloth ratio.

The equations presented above show how the factors considered were found to influence the properties of PBSL beams in the particular series of the tests, and could be used as reference for further study.

## 4. Conclusions

This paper conducted both experimental and analytical studies on 13 beam specimens to study AFRP reinforcement on parallel bamboo strand lumber beams. Strain gauges and Laser Displacement Sensors were used for the tests. The following conclusions can be drawn:

1. Typical bending failure happened for all four PBSL beam specimens without AFRP. The first crack always appeared on the bottom surfaces in the pure bending area for the beams. Natural bamboo joints are the main reason for the first cracks.
2. There are three typical failure modes for AFRP parallel bamboo strand lumber beams. The first failure mode is that both the bamboo fiber and AFRP cloth were pulled off at the bottom of the bamboo beam. As for the failure mode II, besides the split of bamboo fiber, cracks appeared longitudinally along the length of AFRP cloth without pulling off. But, for failure mode III, bamboo fibers split firstly and then the glue between the beam and AFRP lose effectiveness but AFRP cloth didn’t damage.
3. AFRP can improve the flexural properties of parallel bamboo strand lumber beams effectively. With the increase of the cloth ratio, the ultimate bearing capacity, the stiffness, and the ductility for AFRP PBSL beams increased. When the cloth ratio reaches 0.72%, the stiffness of the beam can be increased by 15%.
4. It can be observed that the uniform strain values for the middle cross section conform to the assumption of the plane section. With the increase of load, the neutral axis for the AFRP PBSL beam moved downwards compared with the PBSL beams. AFRP plays a certain role in bearing tensile force for the beam.

## Figures and Tables

**Table 1:** Main parameters of specimen.

Group	*l*/mm	FRP	*n*	Number	*b*/mm	*h*/mm	*λ*
NAP	1860	—	—	4	80	100	5.8
LAP1	1860	AFRP	1	3	80	100	5.8
LAP2	1860	AFRP	2	3	80	100	5.8
LAP3	1860	AFRP	3	3	80	100	5.8

Note: *l* is the length of the specimens; *n* is the Number of sticker layers; *b* is the width of the specimens; *λ* is the shear span ratio.

**Table 2:** Test results.

Group	*ρ*	*F*_u_/kN	*w*_u_/mm	*α*	*EI*/kN·m^2^	*μ*_Δ_
NAP-1	—	50.8	41.56	—	166.3	0.82
NAP-2	—	56.8	54.42	—	167.6	0.93
NAP-3	—	56.1	48.43	—	175.8	1.22
NAP-4	—	51.0	47.72	—	164.9	1.21
Average	—	53.7	48.03	—	168.6	1.04
LAP1-1	0.24%	56.0	54..89	4.2%	209.3	1.14
LAP1-2	0.24%	48.3	48.46	−10.0%	137.9	1.07
LAP1-3	0.24%	57.8	55.56	7.6%	174.1	1.15
Average	—	54.0	52.97	0.6%	173.8	1.12
LAP2-1	0.48%	61.6	72.73	14.6%	163.1	1.26
LAP2-2	0.48%	59.7	64.72	11%	160.6	1.12
LAP2-3	0.48%	53.3	61.98	−0.69%	187.5	1.00
Average	—	59.5	66.48	10.8%	170.4	1.13
LAP3-1	0.72%	78.9	65.10	46.9%	220.8	1.18
LAP3-2	0.72%	70.2	58.12	30.8%	184.9	1.21
LAP3-3	0.72%	67.5	70.77	25.7%	177.2	1.25
Average	—	72.2	64.66	34.5%	194.3	1.22

Note: *w*_u_ is the displacement in the middle span corresponding to the ultimate load; *α* = (*F*_u_ − 53.7)/53.7, is the percentage increase for ultimate bearing capacity.

**Table 3:** Mid-span displacement comparison under various loads.

*F*/kN	*w*_0_/mm	*w*_1_/mm	*w*_2_/mm	*w*_3_/mm	*α*_1_	*α*_2_	*α*_3_
10	6.28	5.51	5.24	5.28	12.1%	16.4%	15.8%
20	12.02	11.18	11.17	10.21	7.0%	7.1%	15.0%
30	18.21	17.51	17.77	15.52	3.8%	2.4%	14.8%
40	26.29	25.99	26.21	21.60	1.1%	0.68%	17.8%
50	36.50	36.31	39.2	29.46	0.53%	−0.073%	19.3%

Note: *w*_0_, *w*_1_, *w*_2_, and *w*_3_ are the average mid-span displacement of beam for group NAP, LAP1, LAP2, and LAP3 respectively; α_1_ = (*w*_0_ − *w*_1_)/*w*_0_; α_2_ = (*w*_0_ − *w*_2_)/*w*_0_; α_3_ = (*w*_0_ − *w*_3_)/*w*_0_.

**Table 4:** The neutral axis position for the ultimate load.

Group	*ρ*	*h*_n_/mm	*β*
NAP1	—	43.49	0.43
NAP2	—	45.64	0.46
NAP3	—	48.47	0.48
NAP4	—	42.56	0.43
Average	—	45.04	0.45
LAP1-1	0.24%	42.5	0.43
LAP1-2	0.24%	43.51	0.44
LAP1-3	0.24%	40.62	0.41
Average	—	42.21	0.42
LAP2-1	0.48%	41.06	0.41
LAP2-2	0.48%	43.32	0.43
LAP2-3	0.48%	42.32	0.42
Average	—	42.23	0.42
LAP3-1	0.72%	36.2	0.36
LAP3-2	0.72%	38.6	0.39
LAP3-3	0.72%	38.32	0.38
Average	—	37.71	0.38

Note: *h*_n_ is the neutral axis height of specimens; *β* = *h*_n_/*h* is the neutral axis relative height of specimens.
